# Supplementary material for: Self-poled piezoelectric polymer composites via melt-state energy implantation
Source: Nat Commun. 2024 Jan 27;15:819. doi: 10.1038/s41467-024-45184-4 (PMC10821934; doi:10.1038/s41467-024-45184-4)
Supplement: Supplementary file 1 — Supplementary Information [file 41467_2024_45184_MOESM1_ESM.pdf]

Supplementary Information

Self-poling Piezoelectric Polymer Composites via Melt-state Energy Implantation

Zhao-Xia Huang*[#,1], Lan-Wei Li[#,1], Yun-Zhi Huang[1], Wen-Xu Rao[1], Hao-Wei Jiang[1], Jin Wang[1], Huan-Huan Zhang[1], He-Zhi He[1], Jin-Ping Qu*[,1]

[1]National Engineering Research Center of Novel Equipment for Polymer Processing, Key Laboratory of Polymer Processing Engineering, Ministry of Education, Guangdong Provincial Key Laboratory of Technique and Equipment for Macromolecular Advanced Manufacturing, Department of Mechanical and Automotive Engineering, South China University of Technology, Guangzhou, 510641 China

Corresponding author: mehuangzx@scut.edu.cn, jpqu@scut.edu.cn

# These authors contributed equally to this work.

Supplementary Text

Phase fraction determination of PVDF

The fraction of β-phase (F(β)) can be calculated using the following equation[1]:

$$F(\beta)=\frac{A_\beta}{\left(\frac{K_\beta}{K_\alpha}\right)A_\alpha+A_\beta}\times 100\% \quad (1)$$

Where A_α and A_β are the absorbance at 764 and 840 cm^{-1}, respectively. K_α and K_β are the corresponding absorbance coefficients, 6.1×104 and 7.7×104 cm^2 mol^{-1}, respectively.

The fraction of γ-phase (F(γ)) can be calculated using the following equations:

$$F(\gamma)=\frac{A_\gamma}{\left(\frac{K_\gamma}{K_\alpha}\right)A_\alpha+A_\gamma}\times 100\% \quad (2)$$

Where A_α and A_γ are the absorbance at 764 and 832 cm^{-1}, respectively. K_α and K_γ are the corresponding absorbance coefficients, 0.365 and 0.150 μm^{-1}, respectively. F(α) is calculated using 1- F(β)- F(γ).

From these equations, we can see that the characteristic peak of β-phase and γ-phase are 840 cm^{-1} and 832 cm^{-1}, which leads to some confusions in distinguishing them. Thus, XRD is usually employed to provide further information of the crystal structures.

Characterization of crystallinity

Calculated *via* DSC

The degree of crystallinity (χ_c) of CP-PBf and El-PBf samples can be calculated by the following equation:

$$\chi_c = \frac{\Delta H_m}{\Delta H_0} \times 100\% \tag{3}$$

where ΔH_m and ΔH_0 represent melting enthalpies of the present sample and of perfectly crystalline PVDF (104.5 Jg-1)[2, 3], respectively.

Calculated *via* XRD

The variations of crystallinity that was calculated from the following equation based on XRD patterns.

$$\chi_c = \frac{S_c}{S_c + S_a} \times 100\% \tag{4}$$

where, S_c and S_a represent the sum of areas of crystalline parts and amorphous parts in XRD diffraction peaks, respectively. Peak separation and area calculation were all done through JADE[4].

Characterization of d33, g33 and FOM

Measurement methods

Method 1:

PBf sample was used to measure piezoelectric properties. First, we define the two faces of PBf as A and B faces, respectively. Sticking Al electrodes on the A and B surfaces respectively, and then putting a layer of Pi tape on the outside of the sample. Second, the PBf sample with A face up is affixed to the baffle, and the positive and negative electrodes of the source meter are connected to the Al electrode near the hammerhead and away from the hammerhead respectively. Then a certain distance is set between the hammerhead and the PBf sample, and the voltage, current and charge values varying with the force are obtained by applying different forces. Third, attach the PBf sample with B side up to the baffle. Through the same steps, connect the b-facing up PBf sample to the positive and negative poles of the source meter. Separately measure voltage, current and charge values under different forces.

Method 2:

The preparation of sample for measurement is same as the method 1. Sticking Al electrodes on the A and B surfaces respectively, and then putting a layer of Pi tape on the outside of the sample. The PBf sample is affixed to the baffle, and the positive and negative electrodes of the source meter are connected to the Al electrode near the hammerhead and away from the hammerhead respectively. Then the hammerhead was controlled move with the force applied on the surface of sample raised from ~2N to a setting value and then drop back to ~2N. During measurement, the force and charge values were recorded. And the slope of the charge *vs* force variations was d33 value.

Calculation details

Here, we define Qp as the piezoelectric charge transfer that induced by PBf sample at a certain force. Through the compressed balance analysis (CBA) method proposed by Zhonglin Wang et al[5], it is known that in the above-mentioned stage, triboelectric charges and piezoelectric charges are saturated in the compressed stage as well as an electrostatic balance is formed.

Specifically, there is an electrostatic balance between triboelectric charges and piezoelectric charges at the compressed stage. Q2 is the transferred charge in the Al electrodes, - Q1 is then

transferred charge in the Kapton layer, q is the total charge transfer in the electrode which contains both piezoelectric and triboelectric parts, Qp is the induced piezoelectric charge. Therefore, the electrostatic balance relationship formed by the PBf sample with the A face up is:

$$Q_2 - Q_1 + q - Q_p = 0 \tag{5}$$

After flipping the device, the electrostatic balance on B side becomes:

$$Q_2 - Q_1 - q' - Q_p = 0 \tag{6}$$

Where $-q'$ is the total charge transfer in the electrode.

By making Eq. (5) - Eq. (6):

$$Q_p = \frac{q + q'}{2} \tag{7}$$

The total induced piezoelectric charge Qp can be calculated by substituting the values of q and q' into expression without considering the interference from triboelectric charges.

The piezoelectric (d33) and piezoelectric voltage (g33) coefficient are obtained by Eq. (8) and Eq. (9), respectively:

$$\mathrm{d}_{33} = \frac{\mathrm{Q_p}}{F} \tag{8}$$

Where Qp is the induced piezoelectric charge and F is the applied force.

$$\mathrm{g}_{33} = \frac{\mathrm{d}_{33}}{\varepsilon_0 \kappa_{33}} \tag{9}$$

Where $\varepsilon_0 = 8.85 \times 10^{-12}$ F/m is the vacuum permittivity and κ_{33} is relative permittivity. The product of d33 and g33 is generally considered to represent the Figof merit (FOM) for piezoelectric energy harvesters and sensors, which can be read as follows:

$$FOM = d_{33} \times \mathrm{g}_{33} \tag{10}$$

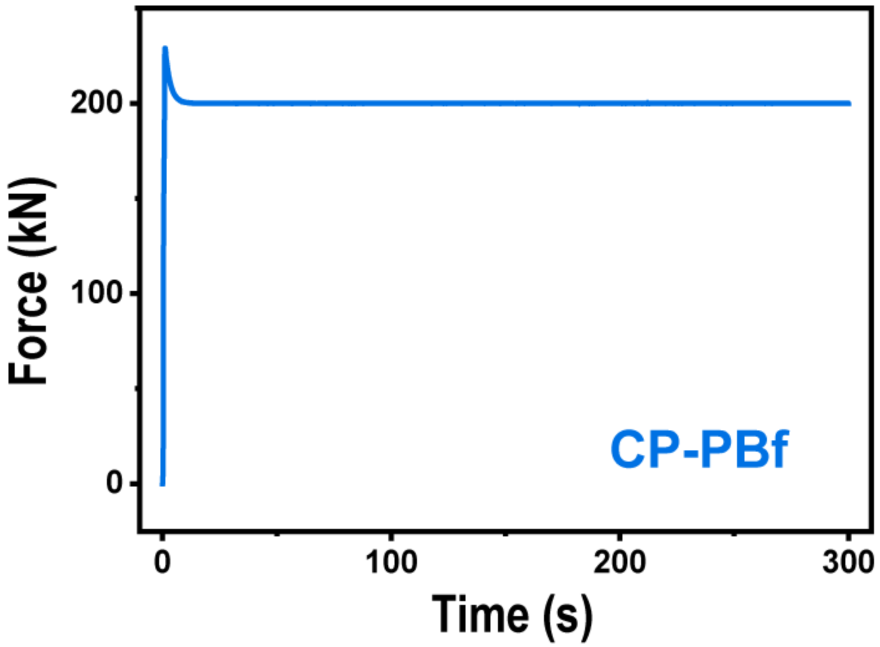

Supplementary Fig. 1. Pressure-time relationship for compression molding CP-PBf.

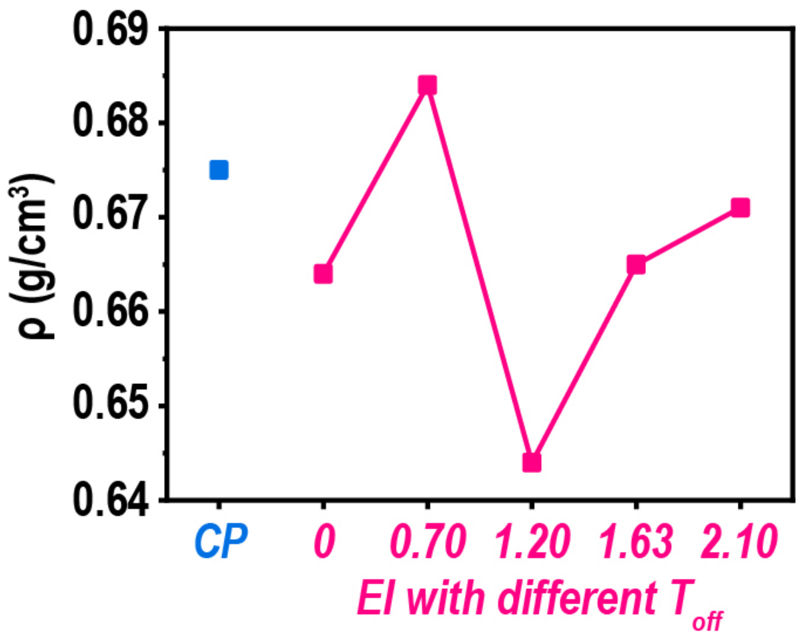

Supplementary Fig. 2. Densities of all PBfs fabricated in this work.

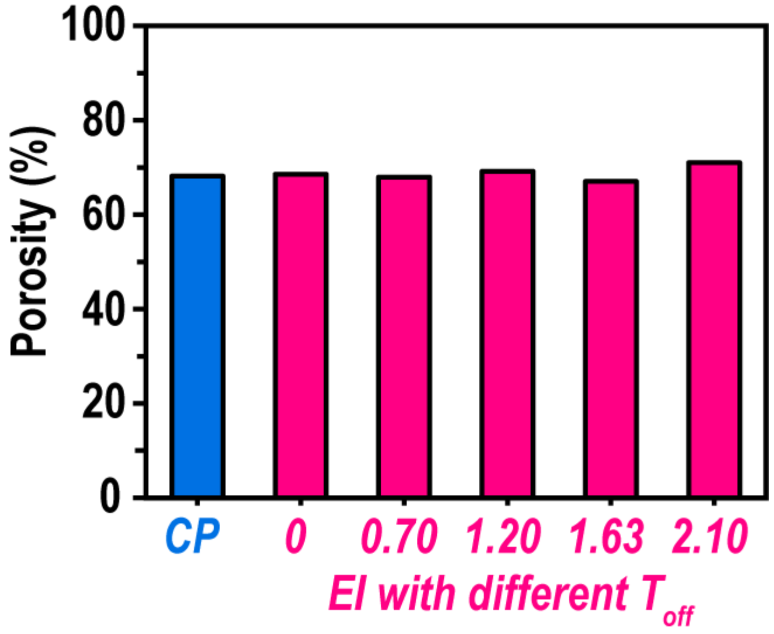

Supplementary Fig. 3. Porosities of all PBfs fabricated in this work.

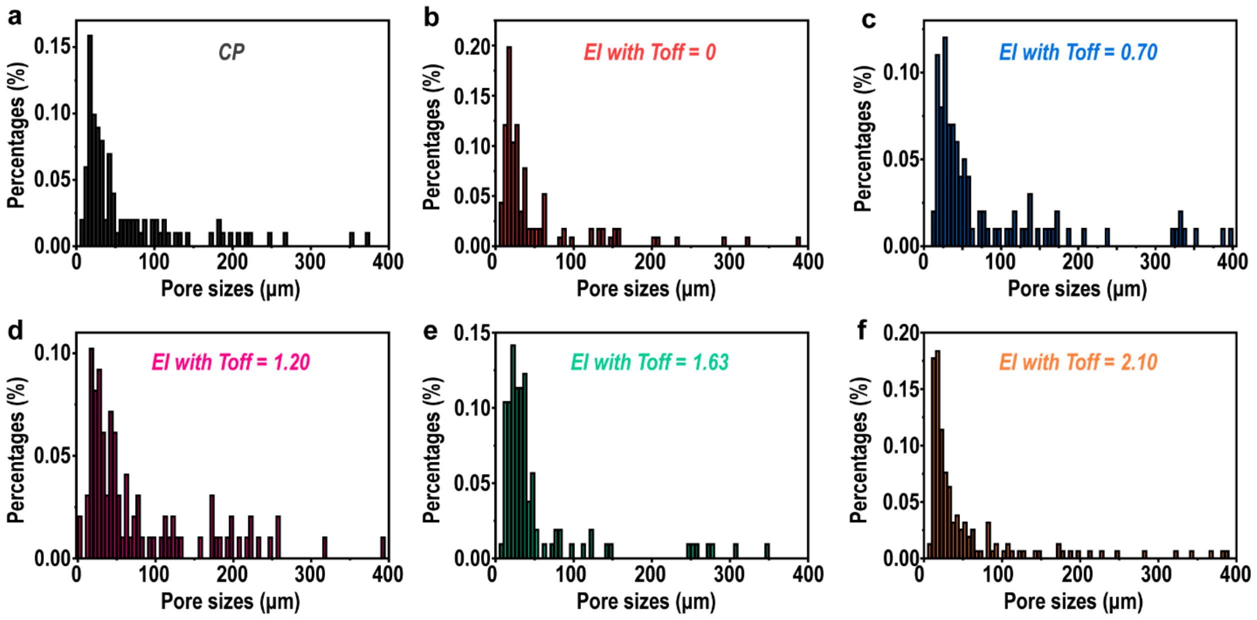

Supplementary Fig. 4. Pore sizes of all PBfs fabricated in this work. The distributions of pore size of PBfs fabricated with different Toff, (a) CP, (b) Toff = 0, (c) Toff = 0.70, (d) Toff = 1.20, (e) Toff = 1.63, (f) Toff = 2.10.

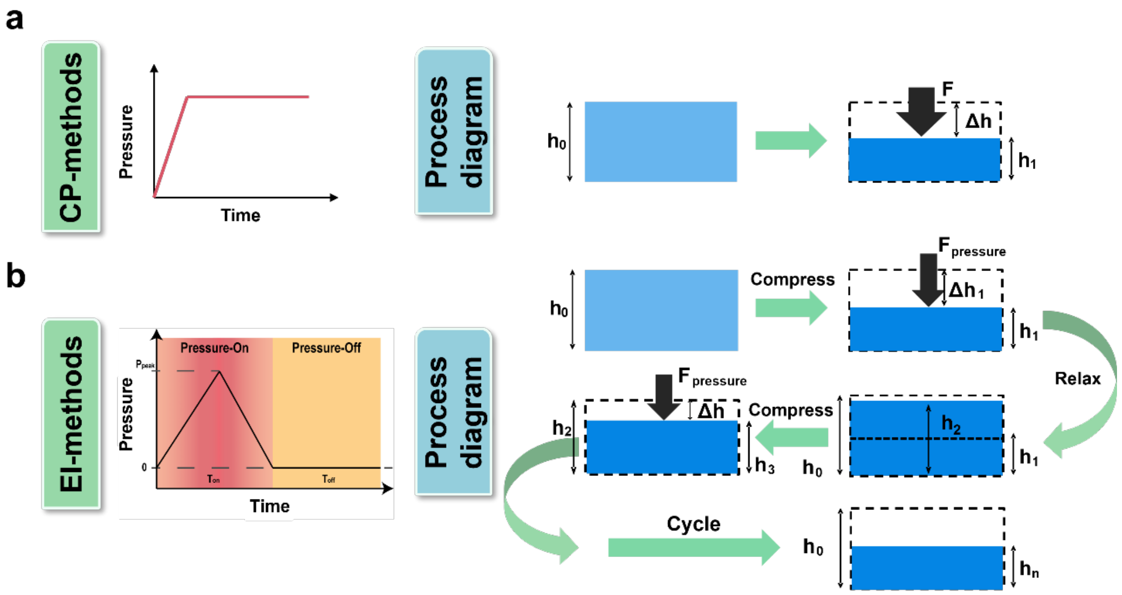

Supplementary Fig. 5. Illustration of the different response of materials under (a) CP- and (b) EI-methods.

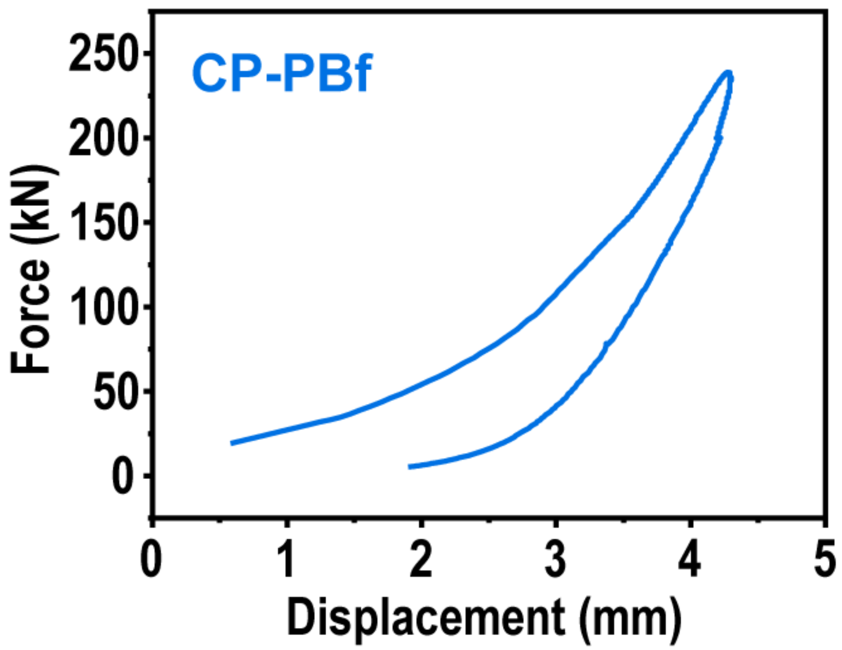

Supplementary Fig. 6. Compression force-delta thickness relationship of CP-PBf.

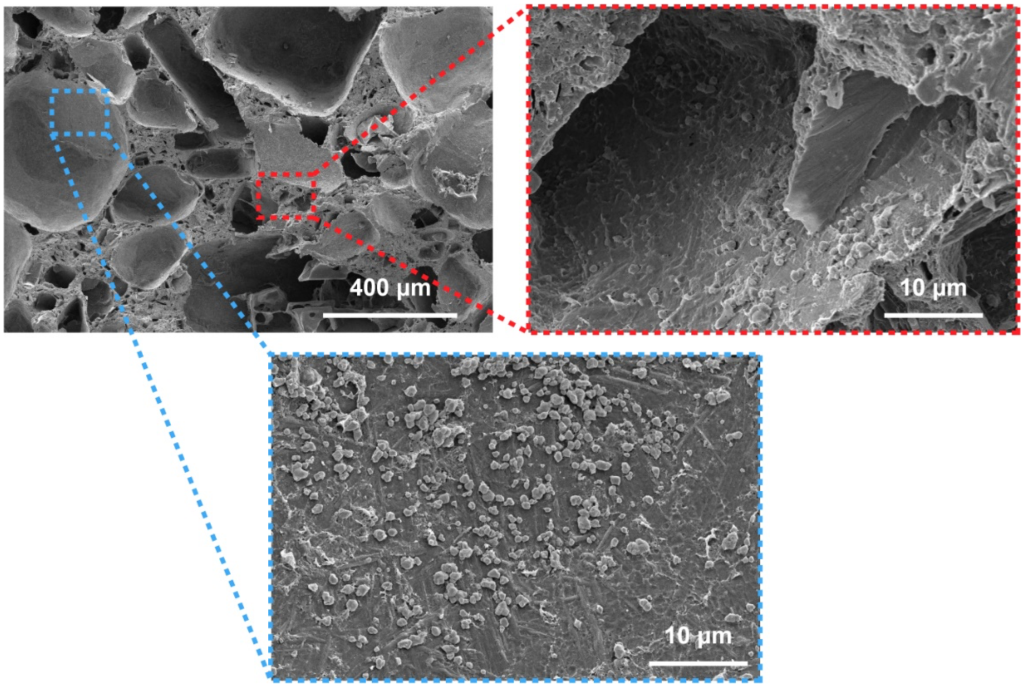

Supplementary Fig. 7. SEM images of CP-PBf.

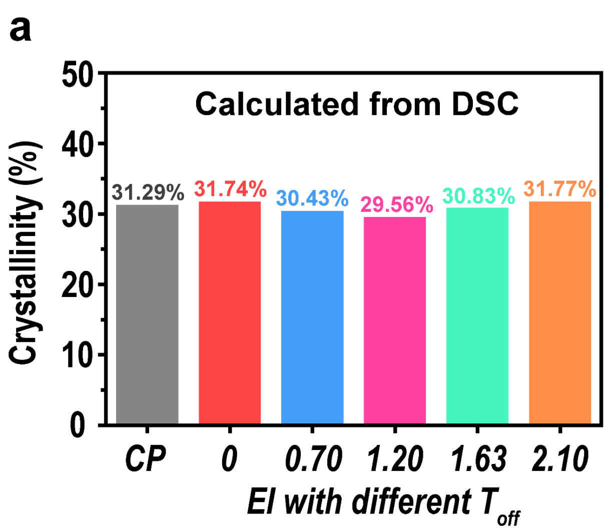

Supplementary Fig. 8. Crystallinities of each sample calculated *via* (a) DSC and (b) XRD.

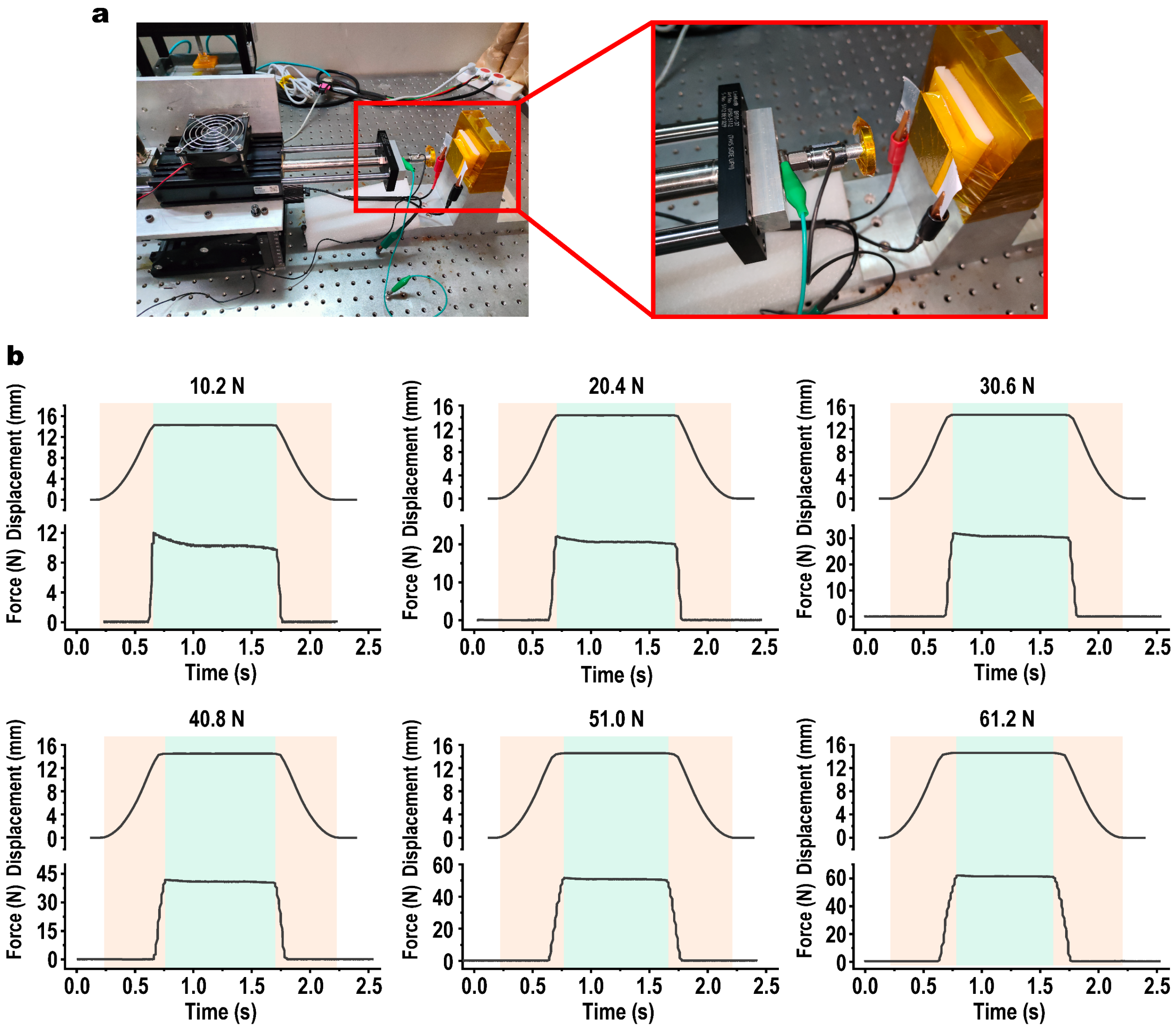

Supplementary Fig. 9. (a) Photographs of the setup for piezoelectric tests, and (b) Applied force-displacement waveform for the piezoelectric tests.

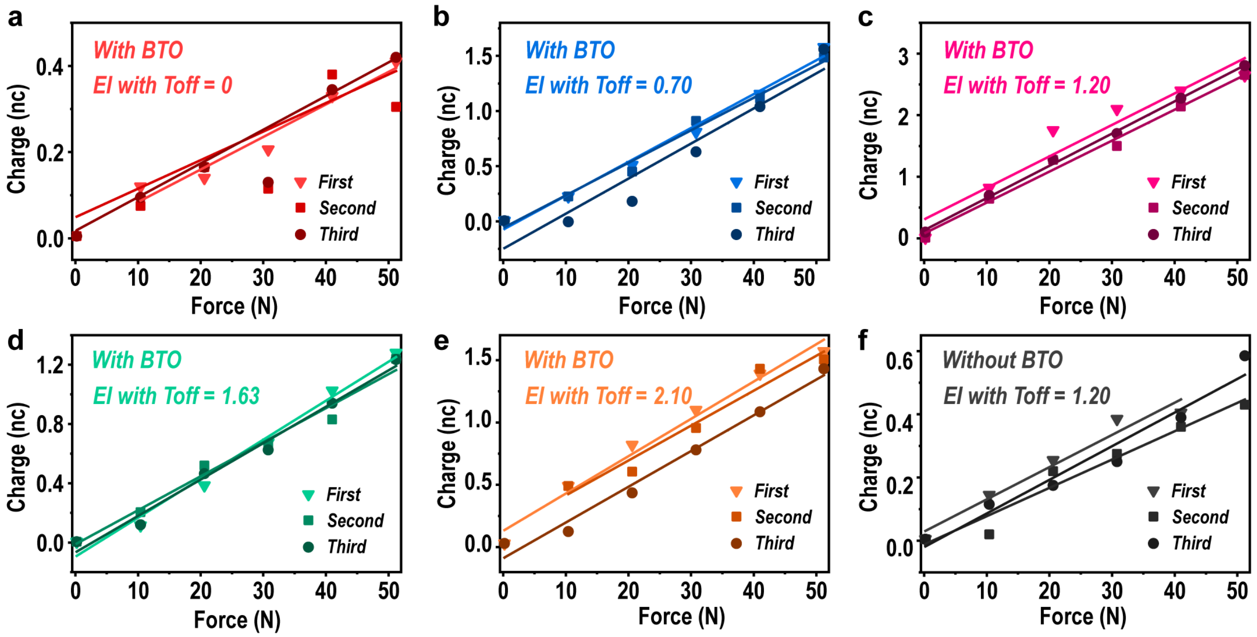

Supplementary Fig. 10. Charge *vs* force relationship of each sample for piezoelectric output measurement. The Charge *vs* force relationship of three repeated tests corresponding to PBfs, (a) Toff = 0, (b) Toff = 0.70, (c) Toff = 1.20, (d) Toff = 1.63, (e) Toff = 2.10. (f) The Charge *vs* force relationship of three repeated tests of Pfs (the BTO free samples) with Toff = 1.20.

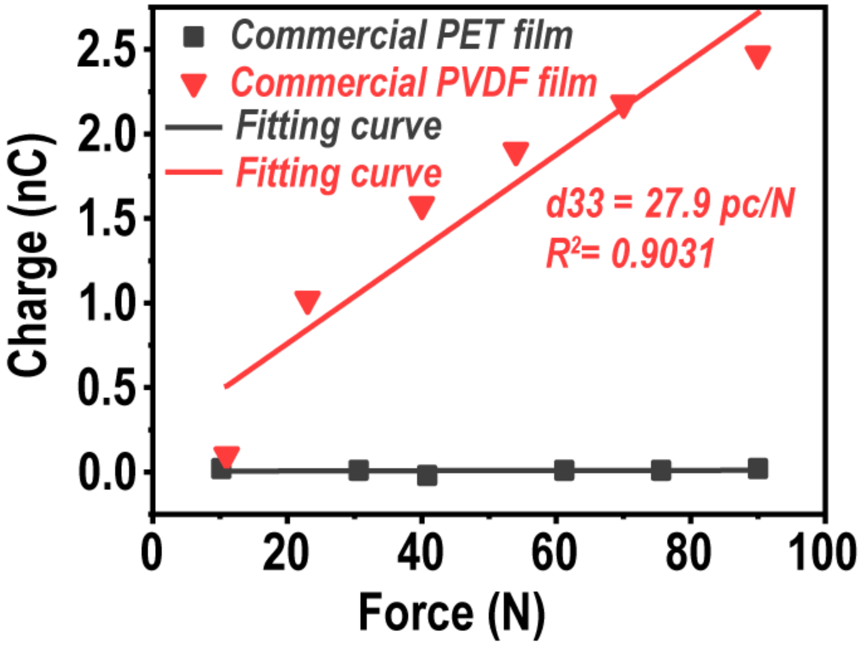

Supplementary Fig. 11. D33 of commercial poled-PVDF film and non-piezoelectric PET film.

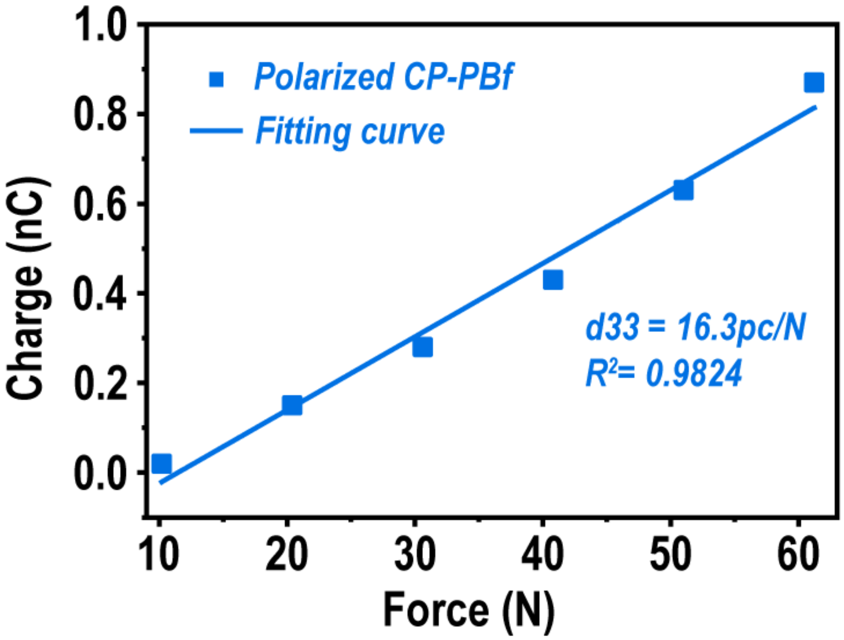

Supplementary Fig. 12. D33 of electrical-poled CP-PBf.

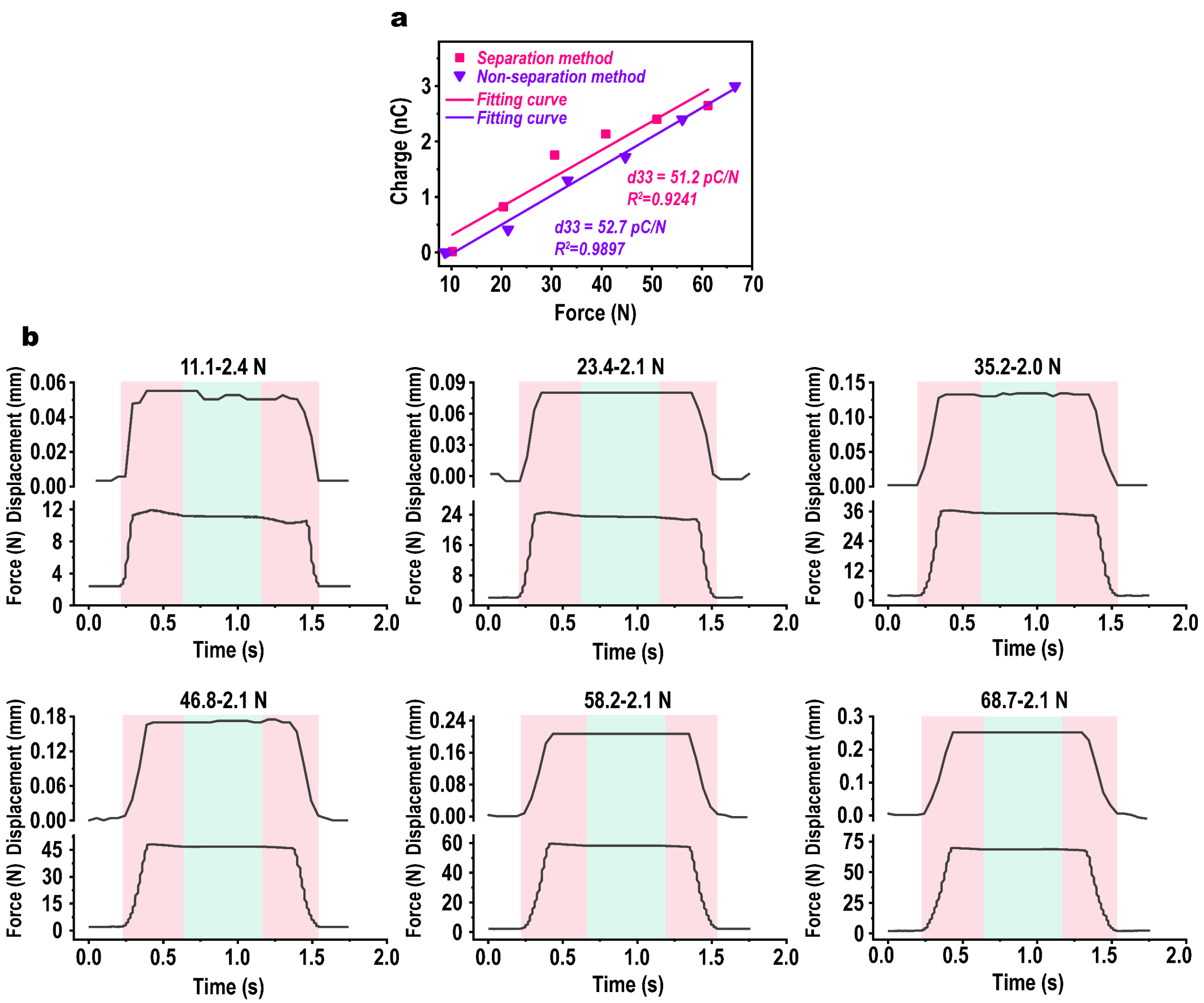

Supplementary Fig. 13. (a) The charge-force relationship of our EI-PBf measured using a separation method and a non-separation method, and (b) applied force / displacement waveform for the non-separation piezoelectric test.

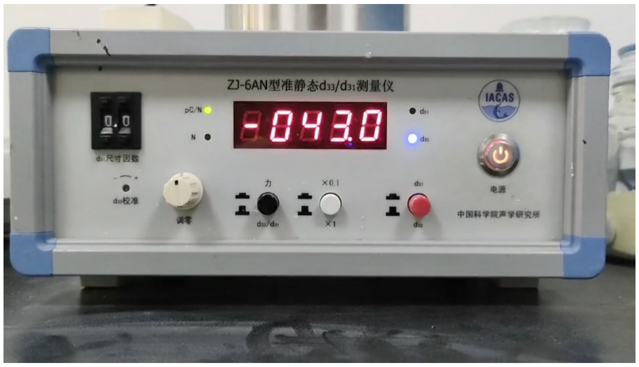

Supplementary Fig. 14. D33 meter test results of EI-PBf sample.

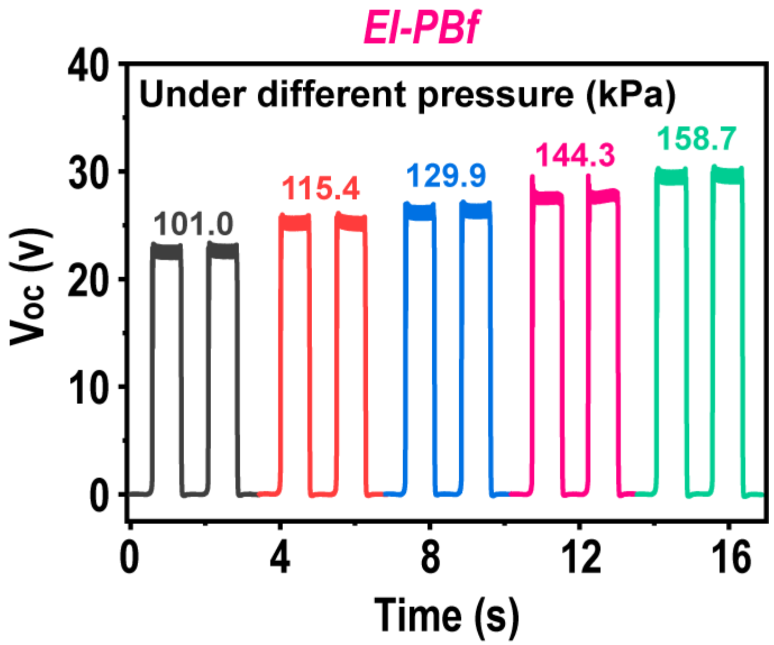

Supplementary Fig. 15. The Voc of EI-PBf under higher applied pressures.

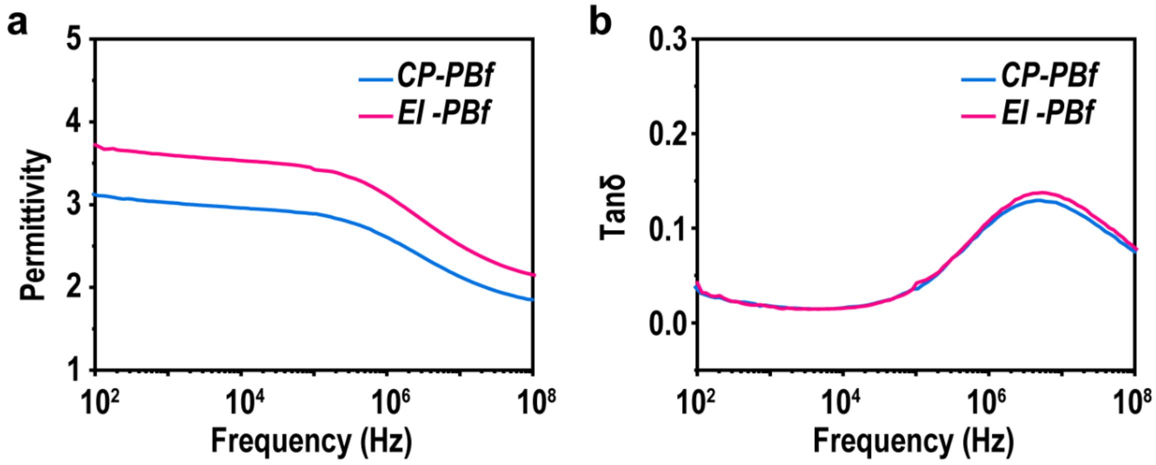

Supplementary Fig. 16. Dielectric properties of CP- and EI-PBfs. Frequency dependence of (a) dielectric constant and (b) dielectric loss tangent of CP-PBF and EI-PBf.

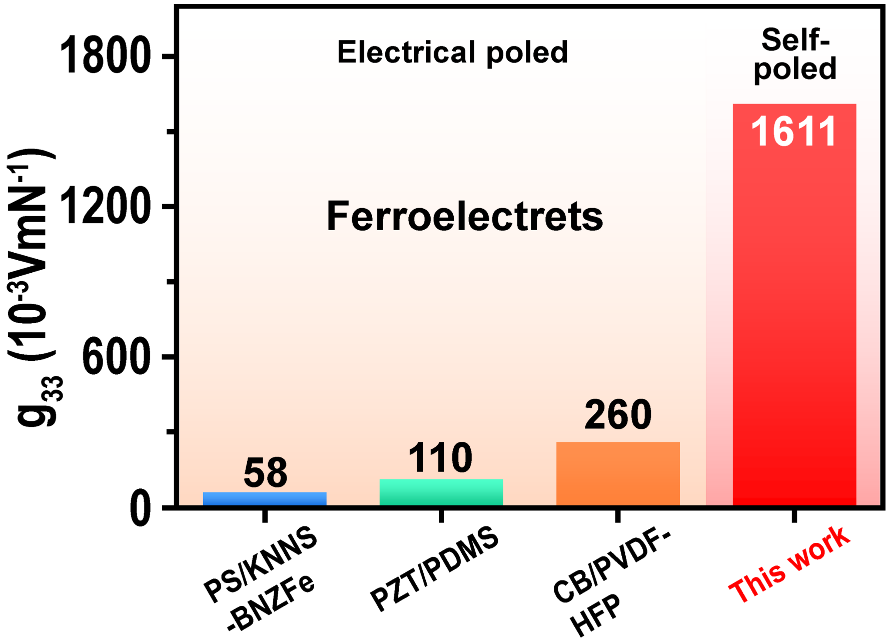

Supplementary Fig. 17. Comparison of the g33 value between our self-poled sample and reported electrical poled ferroelectrets.

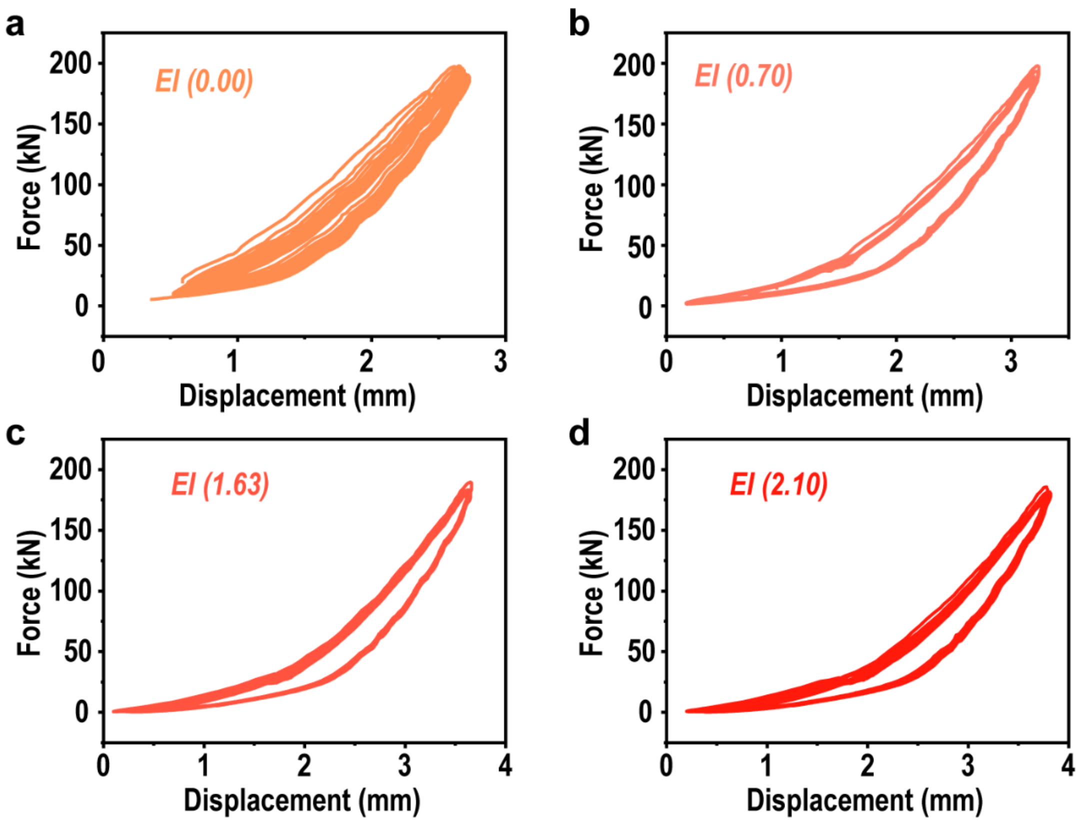

Supplementary Fig. 18. Force-displacement relationships of EI-PBf with various Toff. The Force-displacement relationships of 119 repeated machining operations corresponding to PBfs, (a) Toff = 0, (b) Toff = 0.70, (c) Toff = 1.63, (d) Toff = 2.10.

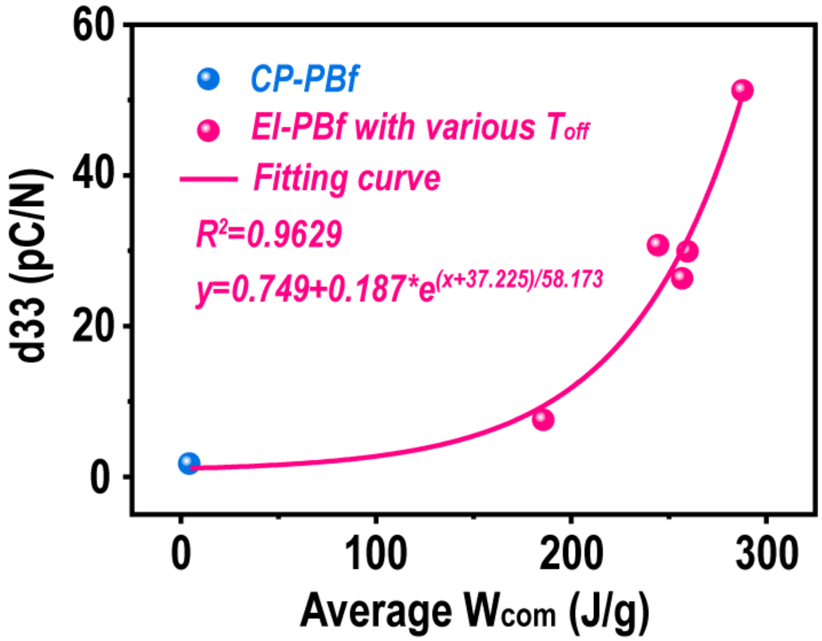

Supplementary Fig. 19. The functional relationship between d33 and average Wcom in CP-PBf and EI-PBf with various T_{off}.

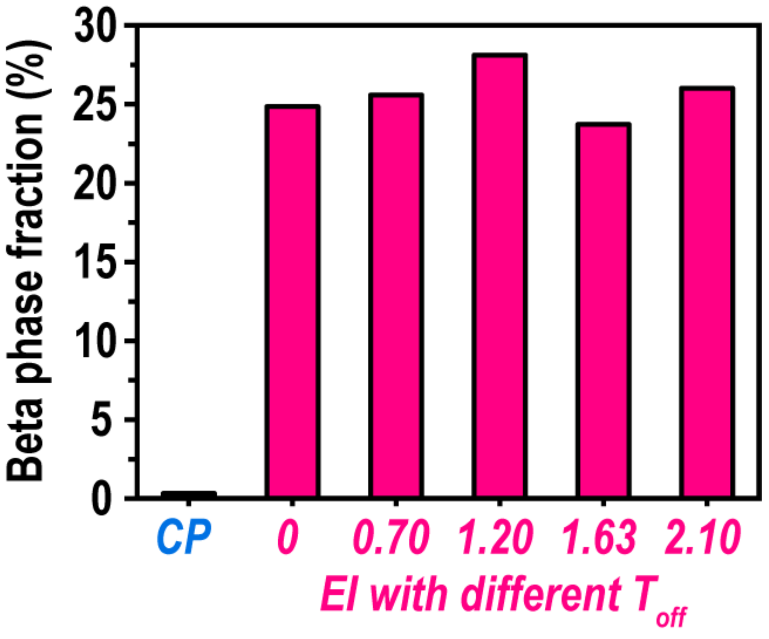

Supplementary Fig. 20. The β-phase of each sample with various T_{off}.

Supplementary Table 1. The d33, g33 and FOM of CP- and EI-PBf compared with other inorganic crystals and ceramics, polymers, organic crystals and hybrid crystals.

	Compound	d33(pC/N)	g33 (10-3 VmN-1)	g_{33}^{ds}(m·VPa-1g-1)	FOM (10-15 m2/N)	Reference
Composite materials	PVDF/BiTiO3	51.2	1611	25020	198105	This work
	Ti3C2Tx/Sm-PMN-PT/PVDF	65	253	1950	16462	[6]
	PVDF-TrFE-Ti3C2Tx	52	402	2700	20904	[7]
	PZT/PDMS	78	881	-	68718	[8]
	PZT/Resin	29	37	-	1073	[9]
Hybrids	(ATHP)2PbBr4	76	660	2600	50160	[10]
	TMBM-MnBr3	112	1120	-	125440	[11]
	TMCM-GaCl4	226	1318	-	297868	[12]
	TMCM-MnCl3	185	1681	9160	310985	[13]
polymers	PVDF	33	310	-	10254	[14]
	BOPVDF	62	359	1970	22274	[15]
	P(VDFTrFE)	25	237	-	5934	[16]
	(β-CN)APB/ODPA	16.5	533	-	8789	[17]
Inorganics	BaTiO3	191	11	-	2172	[18]
	PbTiO3	143	129	-	19367	[19]
	PIN-PMN-Pt	1100	33	-	36949	[20]
	PZT-5A	440	25	-	11000	[21]

Supplementary Table 2. Piezoelectric properties of our EI-PBf and other reported ferroelectrets materials.

Sample	d33 (pc/N)	g33(10^{-3} VmN^{-1})	FOM (10^{-15} m^{2}N^{-1})	References
PS/KNNS-BNZFe	400	58	20887	[22]
PZT/PDMS	497	110	54218	[23]
CB/PVDF-HFP	58	260	15080	[24]
This work	51	1611	82541	

Supplementary Table 3. Crystal data and structure refinement of CP- and EI-PBf.

Compound	CP-PBf	EI-PBf
Formula	PVDF	PVDF/BaTiO3
System	Tetragonal	Tetragonal
Space group	P4mm	P4mm
a (Å)	3.99311	4.00073
b (Å)	3.99311	4.00073
c (Å)	4.03457	4.04584
α (°)	90	90
β(°)	90	90
γ (°)	90	90
V (Å^3)	64.331	64.757
Ti (Å)	0.51848	0.43668
O1 (Å)	0.10908	0.10224
O2 (Å)	0.59701	0.45601
Rp	5.35	6.31
Rwp	7.09	8.33
Chi2	3.82	5.63

Supplementary reference

1. Meng, N.; Ren, X.; Santagiuliana, G.; Ventura, L.; Zhang, H.; Wu, J.; Yan, H.; Reece, M. J.; Bilotti, E., Ultrahigh beta-phase content poly(vinylidene fluoride) with relaxor-like ferroelectricity for high energy density capacitors. *Nat Commun* **2019,** *10* (1), 4535.
2. Lanceros-Méndez, S.; Mano, J. F.; Costa, A. M.; Schmidt, V. H., FTIR AND DSC STUDIES OF MECHANICALLY DEFORMED β-PVDF FILMS. *Journal of Macromolecular Science, Part B* **2001,** *40* (3-4), 517-527.
3. Liu, Z.; Maréchal, P.; Jérôme, R., D.m.a. and d.s.c. investigations of the β transition of poly(vinylidene fluoride). *Polymer* **1997,** *38* (19), 4925-4929.
4. Zhou, Y.; Liu, W.; Tan, B.; Zhu, C.; Ni, Y.; Fang, L.; Lu, C.; Xu, Z. Crystallinity and β Phase Fraction of PVDF in Biaxially Stretched PVDF/PMMA Films *Polymers* [Online], 2021.
5. Chen, C.; Zhao, S.; Pan, C.; Zi, Y.; Wang, F.; Yang, C.; Wang, Z. L., A method for quantitatively separating the piezoelectric component from the as-received "Piezoelectric" signal. *Nat Commun* **2022,** *13* (1), 1391.
6. Su, Y.; Li, W.; Cheng, X.; Zhou, Y.; Yang, S.; Zhang, X.; Chen, C.; Yang, T.; Pan, H.; Xie, G.; Chen, G.; Zhao, X.; Xiao, X.; Li, B.; Tai, H.; Jiang, Y.; Chen, L. Q.; Li, F.; Chen, J., High-performance piezoelectric composites via beta phase programming. *Nat Commun* **2022,** *13* (1), 4867.
7. Shepelin, N. A.; Sherrell, P. C.; Skountzos, E. N.; Goudeli, E.; Zhang, J.; Lussini, V. C.; Imtiaz, B.; Usman, K. A. S.; Dicinoski, G. W.; Shapter, J. G.; Razal, J. M.; Ellis, A. V., Interfacial piezoelectric polarization locking in printable Ti(3)C(2)T(x) MXene-fluoropolymer composites. *Nat Commun* **2021,** *12* (1), 3171.
8. Sappati, K. K.; Bhadra, S., Flexible Piezoelectric 0–3 PZT-PDMS Thin Film for Tactile Sensing. *IEEE Sensors Journal* **2020,** *20* (9), 4610-4617.
9. Nhuapeng, W.; Tunkasiri, T., Properties of 0-3 Lead Zirconate Titanate-Polymer Composites Prepared in a Centrifuge. *Journal of the American Ceramic Society* **2004,** *85* (3), 700-702.
10. Chen, X. G.; Song, X. J.; Zhang, Z. X.; Li, P. F.; Ge, J. Z.; Tang, Y. Y.; Gao, J. X.; Zhang, W. Y.; Fu, D. W.; You, Y. M.; Xiong, R. G., Two-Dimensional Layered Perovskite Ferroelectric with Giant Piezoelectric Voltage Coefficient. *J Am Chem Soc* **2020,** *142* (2), 1077-1082.
11. Liao, W. Q.; Tang, Y. Y.; Li, P. F.; You, Y. M.; Xiong, R. G., Large Piezoelectric Effect in

a Lead-Free Molecular Ferroelectric Thin Film. *J Am Chem Soc* **2017,** *139* (49), 18071-18077.
12. Wang, B.; Hong, J.; Yang, Y.; Zhao, H.; Long, L.; Zheng, L., Achievement of a giant piezoelectric coefficient and piezoelectric voltage coefficient through plastic molecular-based ferroelectric materials. *Matter* **2022,** *5* (4), 1296-1304.
13. Yu-Meng You, W.-Q. L., † Dewei Zhao,† ; Heng-Yun Ye; Yi Zhang; Qionghua Zhou; Xianghong Niu; Jinlan Wang; Peng-Fei Li; Da-Wei Fu; Zheming Wang; Song Gao; Kunlun Yang; Jun-Ming Liu; Jiangyu Li; Song Gao; Kunlun Yang; Jun-Ming Liu; Jiangyu Li; Yanfa Yan; Xiong, R.-G., An organic-inorganic perovskite ferroelectric with large piezoelectric response. *Science* **2017,** *357*, 306–309
14. R. Xu*, S. G. K., Figures of merits of pizeoelectric materials in energy harvesters. In *PowerMEMS*, 2012.
15. Huang, Y.; Rui, G.; Li, Q.; Allahyarov, E.; Li, R.; Fukuto, M.; Zhong, G. J.; Xu, J. Z.; Li, Z. M.; Taylor, P. L.; Zhu, L., Enhanced piezoelectricity from highly polarizable oriented amorphous fractions in biaxially oriented poly(vinylidene fluoride) with pure beta crystals. *Nat Commun* **2021,** *12* (1), 675.
16. Sun, Q.; Xia, W.; Liu, Y.; Ren, P.; Tian, X.; Hu, T., The Dependence of Acoustic Emission Performance on the Crystal Structures, Dielectric, Ferroelectric, and Piezoelectric Properties of the P(VDF-TrFE) Sensors. *IEEE Trans Ultrason Ferroelectr Freq Control* **2020,** *67* (5), 975-983.
17. Park, C.; Ounaies, Z.; Wise, K. E.; Harrison, J. S., In situ poling and imidization of amorphous piezoelectric polyimides. *Polymer* **2004,** *45* (16), 5417-5425.
18. Newnham, R. E.; Bowen, L. J.; Klicker, K. A.; Cross, L. E., Composite piezoelectric transducers. *Materials & Design* **1980,** *2* (2), 93-106.
19. Yan, Y.; Zhou, J. E.; Maurya, D.; Wang, Y. U.; Priya, S., Giant piezoelectric voltage coefficient in grain-oriented modified PbTiO(3) material. *Nat Commun* **2016,** *7*, 13089.
20. Li, G.; Tian, F.; Gao, X.; Tian, H.; Qiao, L.; Liu, J.; Li, F.; Xu, Z., Investigation of High-Power Properties of PIN-PMN-PT Relaxor-Based Ferroelectric Single Crystals and PZT-4 Piezoelectric Ceramics. *IEEE Trans Ultrason Ferroelectr Freq Control* **2020,** *67* (8), 1641-1646.
21. Zhang, Y.; Wang, S.; Liu, D. a.; Zhang, Q.; Wang, W.; Ren, B.; Zhao, X.; Luo, H., Fabrication of angle beam two-element ultrasonic transducers with PMN–PT single crystal and PMN–PT/epoxy 1–3 composite for NDE applications. *Sensors and Actuators A: Physical* **2011,** *168* (1), 223-228.
22. Xue, H.; Jiang, L.; Lu, G.; Wu, J., Multilevel Structure Engineered Lead-Free

Piezoceramics Enabling Breakthrough in Energy Harvesting Performance for Bioelectronics. *Advanced Functional Materials* **2023,** *33* (11), 2212110.

23. Xu, Q.; Wang, Z.; Zhong, J.; Yan, M.; Zhao, S.; Gong, J.; Feng, K.; Zhang, J.; Zhou, K.; Xie, J.; Xie, H.; Zhang, D.; Zhang, Y.; Bowen, C., Construction of Flexible Piezoceramic Array with Ultrahigh Piezoelectricity via a Hierarchical Design Strategy. *Advanced Functional Materials* **2023,** *33* (41), 2304402.

24. Li, Y.; Tong, W.; Yang, J.; Wang, Z.; Wang, D.; An, Q.; Zhang, Y., Electrode-free piezoelectric nanogenerator based on carbon black/polyvinylidene fluoride–hexafluoropropylene composite achieved via interface polarization effect. *Chemical Engineering Journal* **2023,** *457*, 141356.
